# Prostatic Abscess Due to Dual Infection With Mycobacterium tuberculosis and Stenotrophomonas maltophilia

**DOI:** 10.7759/cureus.99858

**Published:** 2025-12-22

**Authors:** Sriram V Raju, Senthil Narayanasamy, Suja Lakshmanan

**Affiliations:** 1 Internal Medicine, Sri Ramachandra Institute of Higher Education and Research, Chennai, IND; 2 General Medicine, Sri Ramachandra Institute of Higher Education and Research, Chennai, IND

**Keywords:** dual infection, genitourinary tb, mycobacterium tuberculosis, prostatic abscess, stenotrophomonas maltophilia infection

## Abstract

Prostatic abscess as an isolated entity is an infrequent and extremely rare manifestation of genitourinary tuberculosis. We report a case of a middle-aged male with type 2 diabetes mellitus and hypertension who presented with persistent lower urinary tract symptoms and fever. Imaging revealed a multiloculated prostatic abscess. Pus culture grew *Stenotrophomonas maltophilia*, while a cartridge-based nucleic acid amplification test confirmed *Mycobacterium tuberculosis*. The patient was managed with appropriate antimicrobial therapy, antitubercular treatment, and supportive care, leading to complete resolution of the abscess. This case underscores the diagnostic challenge posed by dual-pathogen prostatic abscesses and emphasizes the need to consider a tuberculous etiology even in the absence of pulmonary involvement, especially in endemic regions.

## Introduction

Tuberculosis (TB) remains a major global health concern, with India contributing significantly to the global burden [1]. While pulmonary TB is the most common form, extrapulmonary manifestations account for nearly 15-20% of all cases [2], with the genitourinary tract involved in a subset of these. Genitourinary TB (GUTB) accounts for approximately 20% of extrapulmonary TB (EPTB) cases [3]. Among GUTB cases, prostatic involvement is relatively rare, estimated at about 6.6% of all GUTB cases [4], and isolated prostatic TB (i.e., without renal or seminal vesicle disease) is particularly uncommon [5].

Clinically, prostatic TB may mimic chronic prostatitis or a prostatic abscess of bacterial origin and often lacks specific clinical or laboratory features, making the diagnosis challenging [6]. Prostatic abscesses are uncommon and typically caused by Gram-negative bacteria; however, in immunocompromised hosts or individuals with prolonged catheterization or antibiotic exposure, rare organisms such as *Stenotrophomonas maltophilia *may emerge as opportunistic pathogens [7].

We present a rare case of a large prostatic abscess of dual etiology, *Mycobacterium tuberculosis *and *S. maltophilia*. This case highlights the diagnostic complexity and the need for a high clinical suspicion for TB in atypical presentations, even in the absence of pulmonary disease or traditional risk factors.

## Case presentation

A middle-aged male in his 30s from India, with a one-year history of diabetes mellitus and hypertension, presented with complaints of burning micturition and increased urinary frequency for two weeks, along with fever for one week. The fever was high-grade and intermittent and associated with chills, but there was no history of evening rise of temperature. He also reported loss of appetite for one month but denied abdominal pain, chronic cough, or weight loss. There was no history of hematuria, pyuria, urinary incontinence, or urgency.

Despite receiving empirical antibiotics (tablet cefixime 200 mg twice daily for seven days) from a local hospital, his symptoms persisted. There was no significant family history or known contact with TB. He denied smoking, alcohol consumption, high-risk sexual behavior, or other drug intake and had no history of prior catheterization.

On examination, his vital signs were within normal limits. General and systemic examinations were unremarkable. Abdominal examination revealed renal angle tenderness. Per rectal examination, a tender and boggy prostate was shown. Table 1 summarizes the routine baseline investigations performed for the patient.

**Table 1:** Basic blood workup of the patient

Test/parameter	Result	Reference range
Complete blood count		
Hemoglobin	8.2 g/dL	13-17 g/dL (M)
Total leukocyte count	11,900 /mm³	4,000-10,000 /mm³
Neutrophils	86%	40-75%
Lymphocytes	16%	20-40%
Eosinophils	1.20%	1-4%
Platelet count	398,000 /mm³	150,000-450,000 /mm³
Serum electrolytes		
Sodium	128 mmol/L	135-145 mmol/L
Potassium	4.0 mmol/L	3.5-5.0 mmol/L
Chloride	95 mmol/L	98-107 mmol/L
Bicarbonate	20 mmol/L	22-28 mmol/L
Renal function		
Blood urea nitrogen	20 mg/dL	7-20 mg/dL
Serum creatinine	1.4 mg/dL	0.6-1.2 mg/dL
Liver function tests		
SGOT (aspartate aminotransferase)	10 U/L	10-40 U/L
SGPT (alanine aminotransferase)	8 U/L	7-56 U/L
Alkaline phosphatase	68 U/L	44-147 U/L
Total protein	6.0 g/dL	6.4-8.3 g/dL
Albumin	3.0 g/dL	3.5-5.0 g/dL
Globulin	3.0 g/dL	2.0-3.5 g/dL
Total bilirubin	0.93 mg/dL	0.2-1.2 mg/dL
Direct bilirubin	0.26 mg/dL	0.0-0.3 mg/dL
Indirect bilirubin	0.67 mg/dL	0.2-0.9 mg/dL
Urine routine examination		
Color	Straw yellow	-
pH	5	4.5-8.0
Specific gravity	1.005	1.010-1.030
Glucose	2+	Negative
Protein	Negative	Negative
Leukocytes (WBCs)	3+	Negative
Pus cells	138 /HPF	<5 /HPF
Urine Gram stain	Moderate pus cells, no organisms	-
Diabetes profile		
Random blood sugar	220 mg/dL	<140 mg/dL
HbA1C	10.50%	<5.7% (normal), <7% (target for diabetics)
Inflammatory marker		
Erythrocyte sedimentation rate	48 mm/hr	<20 mm/hr
Other tests		
HIV	Nonreactive	-
Hepatitis B (HBsAg)	Nonreactive	-
Hepatitis C (anti-HCV)	Nonreactive	-
Prostate-specific antigen	22 ng/mL	<4 ng/mL

Blood and urine cultures were sterile despite clinical suspicion of a urinary tract infection. A comprehensive evaluation for fever was conducted, including serologies for dengue, scrub typhus, and leptospirosis, as well as quantitative buffy coat testing for malaria, all of which were negative.

**Figure 1:**
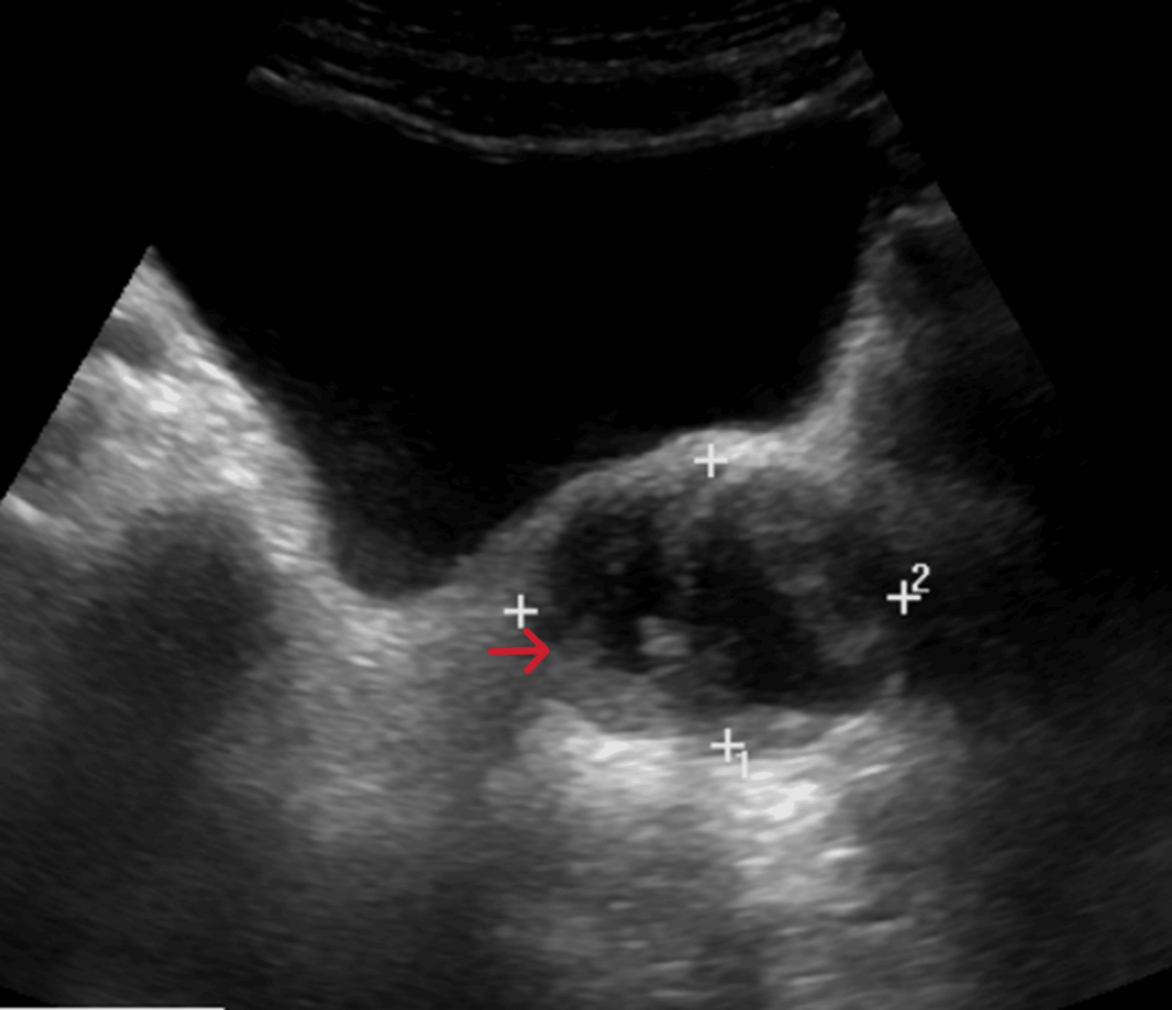
Ultrasound of the abdomen showing an enlarged prostate with a heterogeneous collection

ECG revealed a normal sinus rhythm. Chest radiograph showed increased bronchovascular markings in both lung fields. Abdominal ultrasonography demonstrated features of cystitis and an enlarged prostate (55 cc) with a heterogeneous collection (5.5 × 3.0 × 4.4 cm; volume 35 cc) containing internal debris, suggestive of a prostatic abscess (Figure 1). Two-dimensional echocardiography was unremarkable, with no evidence of vegetations.

A transrectal ultrasound (TRUS)-guided aspiration of the prostatic abscess was performed. Imaging revealed a heterogeneous multiloculated collection (3.5 × 2.7 × 5.0 cm; volume 25 cc) replacing most of the prostate, with increased peripheral vascularity noted in the collection. An additional ill-defined collection (2.2 × 0.5 × 1.7 cm; volume 1 cc) near the bladder neck of the prostate was also observed.

Pus culture from the aspirate grew *S. maltophilia*, which was susceptible to cefoperazone-sulbactam. Cartridge-based nucleic acid amplification test (CBNAAT) of the abscess material was performed due to the endemicity of TB in India, and it tested positive for *M. tuberculosis*. Acid-fast bacilli (AFB) and fungal stains were negative. Histopathological analysis of the aspirated pus revealed acute suppurative inflammation with no malignant cells. Sputum AFB smear and GeneXpert MTB/RIF were also negative. The Mantoux test was positive, showing 16 mm induration after 72 hours. Extended cultures for *Burkholderia *were negative. A contrast-enhanced CT (CECT) of the abdomen was planned to evaluate for other abscesses, but the patient declined due to logistic issues.

The patient was started on intravenous cefoperazone-sulbactam (2 + 1 g) twice daily for 14 days, along with weight-based antitubercular therapy (ATT) for six months. This included a two-month intensive phase with rifampicin 600 mg OD, isoniazid 300 mg OD, pyrazinamide 1500 mg OD, and ethambutol 1200 mg OD, followed by a four-month continuation phase with rifampicin 600 mg OD and isoniazid 300 mg OD. Parenteral iron supplementation was administered to correct iron deficiency anemia.

**Figure 2:**
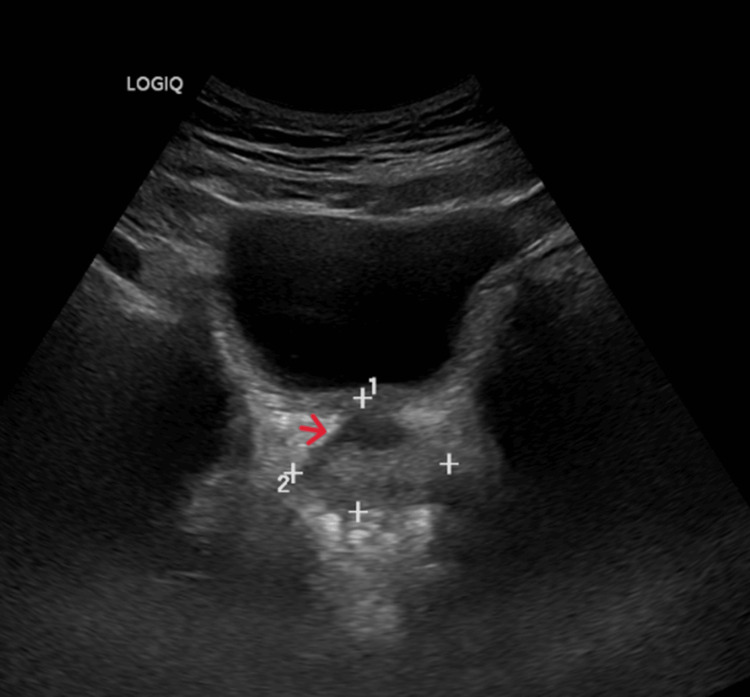
Repeat ultrasound of the abdomen showing no residual prostatic abscess

On follow-up, the patient tolerated ATT well and reported symptomatic relief. Repeat abdominal ultrasonography after two months showed only minimal residual prostatic abscess (Figure 2).

## Discussion

TB remains a major global health concern, with an estimated 10.8 million new cases worldwide in 2023 [1]. While the majority of cases involve the lungs, EPTB accounts for approximately 15-20% of all cases [2,3]. GUTB comprises around 20% of EPTB cases [3]. India continues to have the largest absolute TB burden, contributing roughly a quarter of global cases and reporting approximately 2.6 million notified TB cases in 2024.

TB of the prostate is a rare entity among GUTB cases, constituting roughly 6.6% of GUTB [4]. It is more commonly seen in association with pulmonary or renal TB and most often affects immunocompromised individuals. Isolated prostatic involvement, particularly in immunocompetent or minimally immunocompromised hosts, is uncommon and frequently underdiagnosed [5,8]. Clinical presentation of GUTB is variable: patients may present with constitutional symptoms, sterile pyuria, recurrent urinary tract infections, hematuria, voiding dysfunction, or, less commonly, focal organ complications such as renal cavitation, ureteric strictures, or prostatic abscesses. Prostatic TB is an uncommon manifestation, often diagnosed incidentally or when complications (abscess, urinary retention, or a suspicious prostatic lesion) prompt evaluation. Published case series and reviews emphasize that prostate involvement is rare and is frequently identified either on histology after transurethral resection or via tissue sampling when GUTB is suspected.

Definitive diagnosis of GUTB should be pursued using microbiological tests whenever possible. Nucleic acid amplification tests (e.g., CBNAAT/Xpert MTB/RIF) and mycobacterial culture from appropriate specimens are recommended, while imaging (USG/CT/MRI/TRUS) and histopathology serve as complementary tools. Management follows standard ATT for EPTB (2HRZE/4HR for drug-sensitive cases), with drainage or surgery reserved for complications and to obtain diagnostic material. Outcomes are generally favorable when a diagnosis is made early and ATT is started promptly, but delayed diagnosis may lead to structural complications requiring urological interventions.

In our case, the absence of pulmonary symptoms and negative sputum studies supports isolated prostatic involvement. Diagnostic confirmation was established through CBNAAT of aspirated pus, a rapid and highly sensitive method for detecting *M. tuberculosis *even in extrapulmonary specimens (per institutional protocol). Histopathology showed acute inflammation but lacked granulomas or AFB, likely reflecting sampling variability or the early suppurative stage of infection [6,9].

Prostatic abscesses are uncommon in the antibiotic era and most often result from inadequately treated acute bacterial prostatitis. They are typically caused by Gram-negative bacilli, with *Escherichia coli *being the predominant pathogen, followed by *Klebsiella *spp., *Pseudomonas aeruginosa*, and occasionally Gram-positive organisms [10]. Risk factors include uncontrolled diabetes mellitus, chronic urinary retention, prolonged catheterization, and other causes of immunosuppression. Clinically, patients may present with fever, dysuria, urinary retention, and a tender, fluctuant prostate on digital rectal examination. Imaging, usually transrectal ultrasonography or CECT, is critical for diagnosis and to guide drainage [6,9].

*S. maltophilia *is increasingly recognized as a cause of urinary tract infections and can act as an opportunistic pathogen, particularly in hospital settings or in patients with prior antibiotic exposure [7]. Its involvement in prostatic abscess is exceedingly rare (with no substantial human series), but given its ability to form biofilms and intrinsic resistance to many broad-spectrum antibiotics (including carbapenems), it represents a therapeutic challenge [7,11]. In our patient, the culture of the abscess aspirate yielded *S. maltophilia *sensitive to cefoperazone-sulbactam, which was used for treatment. Other commonly used agents include trimethoprim-sulfamethoxazole, levofloxacin, and minocycline; therapy should be guided by susceptibility testing due to variable resistance patterns [7,11].

The coexistence of *M. tuberculosis *and *S. maltophilia *in the same abscess is highly unusual and presents unique diagnostic and therapeutic challenges. While *M. tuberculosis* requires prolonged ATT, *S. maltophilia *demands specific antimicrobial coverage due to its resistance profile. Empirical antibiotic therapy alone may be insufficient, making microbiological evaluation of the abscess aspirate essential to guide effective treatment. The presence of *S. maltophilia *should also prompt clinicians to evaluate for underlying immunosuppression, nosocomial exposure, or biofilm-associated infection [7,11].

Management in this case involved a combination of abscess drainage, targeted antibiotic therapy, and a standard antitubercular regimen (2HRZE + 4HR for six months), similar to pulmonary TB, with extension of therapy in selected cases based on clinical judgment. The patient’s favorable clinical response underscores the importance of timely intervention and individualized antimicrobial selection in complex infections. This case emphasizes the need for heightened clinical suspicion for tuberculous etiology in prostatic abscesses, particularly in TB-endemic regions, and the potential for unusual co-pathogens such as *S. maltophilia *in susceptible individuals. Early diagnosis using molecular techniques and culture-based methods, along with appropriate antimicrobial therapy, can lead to complete recovery even in atypical dual infections.

## Conclusions

Prostatic TB is a rare and often overlooked diagnosis, particularly when coexisting with opportunistic pathogens such as *S. maltophilia*. This case highlights the importance of testing for TB in any abscess, even in the absence of pulmonary manifestations, especially in endemic regions. Timely imaging, microbiological evaluation, and molecular diagnostics are essential for accurate diagnosis. Early, targeted therapy combining antitubercular treatment with appropriate antimicrobial agents can lead to complete clinical resolution, even in complex infections involving unusual microbial etiologies.
